# Social media behaviors and body type ideals predict weight loss and food tracking behaviors among recreational climbers

**DOI:** 10.3389/fspor.2024.1408209

**Published:** 2024-06-13

**Authors:** Nicholas Slagel, Katie Kage, Sarah Wichern

**Affiliations:** ^1^ Department of Kinesiology, Nutrition and Dietetics, University of Northern Colorado, Greeley, CO, United States; ^2^ Department of Nutrition, Kinesiology and Health, University of Central Missouri, Warrensburg, MO, United States

**Keywords:** climbing, body image, weight loss, social comparison, social media

## Abstract

**Introduction:**

Elite and recreational climbers may be at risk for disordered eating, low energy availability (LEA), and increased injury as a result. Social media use among athlete and non-athletes can lead to body image disturbances resulting in unhealthy weight loss practices exacerbating LEA and injury risk. Therefore, the objective of this study was to examine relationships between social comparative behaviors on social media, body type ideals and outcomes, and health behaviors among adult recreational climbers.

**Methods:**

Participants (*n* = 324) were adult recreational climbers from the U.S. (29.30 ± 9.99 years old and 50% female). Participants answered a 66-item questionnaire comprised of demographics, climbing characteristics, social media behaviors, body type ideals, training and nutrition-seeking behaviors, and weight and food tracking behaviors.

**Results:**

Most participants (78.7%) indicated strength-to-weight ratio was important for climbing performance. Many participants perceived they could perform better at rock climbing if their body proportions were different (59.3%). These body type ideals were found to be significant predictors of performing weight loss and food-tracking behaviors (all *p* < 0.001). Higher amounts of social comparative behaviors on social media and social physique anxiety independently and significantly predicted attempting weight loss to improve climbing ability (*p* < 0.001 and *p* = 0.001 respectively). Those who followed climbing influencers, used Instagram frequently for training and nutrition information, perceived they could perform better at rock climbing if their body proportions were different, or were female and college-aged had significantly higher mean social comparative behavior scores (all *p* < 0.01).

**Discussion:**

This study expands on prior work with elite climbers by providing a possible explanation for how climbing-related body type ideals and certain social media behaviors can perpetuate negative body image and compensatory behaviors among a general climbing population. Because unhealthy weight management behaviors can lead to injury and health disturbances, broad education programming and social media campaigns should be developed to shift body ideals and nutrition behaviors among recreational climbers.

## 1 Introduction

The rise in popularity of climbing recreationally indoors and outdoors has grown globally after the sport's Tokyo Olympics debut in 2020. With this new attention, research has expanded in scope and begun to characterize physiologic and psychologic aspects of the sport. Research linking the health and performance of elite and recreational climbers has demonstrated dietary habits that were low in energy and carbohydrate availability (1, 2). These two studies show mixed results regarding whether climbing level leads to significantly different levels of energy availability. Related research among elite sport climbers reveals low energy availability (LEA) (3) and moderating factors like disordered eating are prevalent (4), which are associated with a higher prevalence of outcomes like amenorrhea and injury (5, 6). Low EA can exist with or without moderating factors like eating disorders or disordered eating (7).

Psychological pressures to perform at elite levels and body dissatisfaction are individual risk factors for restrictive or disordered dietary behaviors (8), which can moderate the risk and intensity of LEA (9) and therefore injury outcomes (10, 11). A recent meta-analysis revealed that athletes may have less body dissatisfaction than non-athletes and similar levels of eating disorder psychopathology (e.g., drive for thinness, dietary restriction) (12). However, further analysis revealed athletes competing in aesthetic or lean sports had higher levels of body dissatisfaction and eating disorders. This supports earlier research noting lean sport athletes have a higher incidence body dissatisfaction, restrictive eating, eating disorder, and menstrual dysfunction compared to non-lean athletes (13, 14). Additionally, these differences were not seen when comparing elite to non-elite athletes (14). Research that has analyzed climbers’ anthropometric profiles would characterize climbing as a “lean sport” (15), therefore body image disturbances among climbers may be contributing to LEA and its associated health and injury outcomes.

Current research about body image concerns among elite and non-elite climbers is limited, however, those studies that do exist reveal body image concerns do exist among climbing populations. In one study, elite and female climbers felt more pressure to maintain a body type ideal for rock climbing than their recreational counterparts (16). They also noted that the abundance of body-centric presentations of climbing athletes in the media influenced how the respondents interpreted body type ideals in climbing. In a recent digital ethnography of climbing-specific websites and forums, the broader climbing community believes disordered eating and negative body image are prevalent in the community (17). Many climbers in this study believed that a higher strength-weight ratio is beneficial to climbing ability, yet there appeared to be a growing awareness or trend toward countering this ideal. Taken together, these recent studies show that body and fitness ideals do exist in the climbing community. Among the many potential factors that may perpetuate these ideals among climbers is the role media and social media play in creating social norms and ideals around body image, physique, or beauty standards.

Many recent studies have found a link between high amounts of social media usage, body image disturbance, and disordered eating among younger adult populations (18). One theory is that social media can portray a “thin ideal,” and following influencers on social media that portray these ideals leads to negative body image due to social comparison behaviors (19). A recent study on various health and fitness influencers on TikTok found that hashtags about weight-centric topics like “Weight Loss Transformation” or “What-I-Eat-in-a-Day” will garner more views than other hashtags (20). Similarly, fitness and strength content in social media can create a fit ideal that may perpetuate similar body image disturbances as thin ideal and weight-centric content (21). One explanation for how social media content influences the body image of social media users is the role of upward social comparison (22). This research demonstrates that certain users of social media are at a higher risk of comparing themselves and their bodies to their peers and influencers, which is linked to a higher incidence of body dissatisfaction and pathways of eating disorder psychopathology.

Despite research evidence countering the need for climbers to conform to body type and weight normative ideals to improve climbing ability, the fit and thin-ideal content on social media may outweigh this counter-narrative. Studies looking at body image attitudes and associated risks among climbers are emerging, however, most studies focus on dietary, health, and injury risks among elite-level climbers who make up a small proportion of the climbing community. Many factors may explain the risk for body dissatisfaction, like social media use, but this has yet to be characterized among the broader climbing community using validated tools. Therefore, the objective of this study was to examine relationships between social comparative behaviors on social media, social physique anxiety outcomes, and health behaviors among adult recreational climbers.

## 2 Methods

### 2.1 Participants

The participants included adults (≥18 years of age) who were climbing at a private or university climbing gym from July through October of 2022. Institutional Review Board (IRB) approval was granted through the University of Northern Colorado. As an incentive, $10 Amazon e-gift cards were randomly awarded to 200 participants via email following the completion of the study.

A database of private and university rock climbing facilities across the U.S. (*N*  = 540) and their contact information was created from a comprehensive online resource (23). Each gym and university center were separated by their associated U.S. region, then half of the facilities from each region (*n* = 270) were randomly selected to support the researchers in distributing the survey and promotional materials. The selected facilities were emailed instructions for participation with a link to the study survey for email distribution, printable flyers with a QR code linking the survey to be posted in facilities, and e-flyers to be posted on social media and newsletters. Participants were recruited by the partnering facilities’ preferred methods (e.g., flyer with QR code linking survey, email, social media, or newsletter with a survey link). Prior to participation in the survey participants provided digital informed consent to participate.

### 2.2 Questionnaire

Participants answered a 66-item questionnaire in electronic format Qualtrics (Qualtrics XM 2021, Provo UT). The survey was open from July through October of 2022. The questionnaire included six distinct sections with two sections containing subsections. These major sections assessed demographic characteristics, personal climbing characteristics and abilities, social media behaviors, body type ideals, training and nutrition-seeking behaviors, and weight and food tracking behaviors.

#### 2.2.1 Demographic characteristics

Data such as age, region of the U.S., gender identity, race and ethnicity identity, education, annual income, and household structure were collected.

#### 2.2.2 Personal climbing characteristics and abilities

Questions collected information about years of climbing experience, frequency of climbing indoors and outdoors, preferred climbing types, perceived skill level, participation in climbing groups or competitions.

#### 2.2.3 Social media behaviors

Questions asked about use of climbing specific content on social media, how many social media sites were used, which social media sites were used most frequently for climbing content, and whether they follow professional rock climbers or climbing influencers. A subsection of social media behaviors assessed technology-based social comparison and feedback-seeking (Technology-based SCFS). Technology-based SCFS was measured using five subscale items from the Motives for Electronic Interaction—Social Comparison and Feedback Seeking Subscale (MEI-SCFS) (24). Technology-based SCFS behaviors are characterized by how people evaluate themselves while navigating social media. The higher the score the more one compares themselves to others and seeks feedback on social media.

#### 2.2.4 Body type ideals

Three specific questions were developed to capture various aspects of body and weight ideals. Participants were asked to rank how important a high strength-to-weight ratio and body type is for climbing performance. The other measure assessed whether participants perceived their performance would improve if they had different body proportions. A subsection of body type ideals included social physique anxiety (SPA). Social physique anxiety is considered a sub-type of social anxiety that one experiences when they believe they are being observed or judged on their appearance (25). SPA was measured using the Social Physique Anxiety Scale (SPAS) (25). SPAS questions 1–10 were included verbatim, question 11 was excluded, and item 12 was modified to be more relevant for climbing populations. Using 11 items, scores can range from 11 to 55, with higher scores on the scale indicating a higher level of anxiety about their appearance or physique.

#### 2.2.5 Training and nutrition-seeking behaviors

These measures were developed to assess who participants perceive as qualified to provide climbing training and nutrition support, whether they have sought or been given training or nutrition advice for climbing purposes, where or who they sought information from, and how frequently.

#### 2.2.6 Weight and food tracking behaviors

These behaviors included six measures that assessed whether a participant has ever attempted weight loss for climbing purposes, sought a professional for weight loss, the frequency of losing weight for climbing purposes, frequency of self-weighing on a scale, whether one uses food tracking methods and how frequently food tracking methods are used.

### 2.3 Statistical analysis

Results were computed using IBM SPSS Statistics for Macintosh, version 29.0 (IBM Corp., Armond, N.Y., USA). The significance level was set at *p* < 0.05 and confidence intervals were set at 95%. Data was checked for homogeneity of variance using Levene's test, and normality using Shapiro–Wilk's. Descriptive and comparative statistics such as independent *t*-tests and one-way ANOVA were used for exploratory analyses of all measures. When normality assumptions were violated, non-parametric tests (Mann-Whitney or Kruskal-Wallis) were used when appropriate. Based on the original validation studies of the SPAS, questions 2, 5, and 8 were reverse-coded prior to analysis. Cronbach's alpha was used to test the reliability of the MEI-SCFS and SPA scales. Based on relationships noted from descriptive and comparative analyses, logistic regression models were tested to evaluate what variables were predictive of weight and food-tracking behaviors among participants.

## 3 Results

### 3.1 Sample characteristics

There were 446 initial responses to the survey, 122 of which were incomplete, resulting in a final analytic sample of (*n*  =  324). The mean age of the sample was 29.30 ± 9.99 years old. Half (49.7%) of the sample identified as female, (46%) as males, and (4.3%) identified as nonbinary or third gender. The vast majority (88.9%) of the sample identified as White or Caucasian. Most participants (67.9%) considered themselves “Intermediate level” climbers, many (44.4%) have been climbing between 2 and 5 years, and on average climbed indoors 3.00 ± 1.70 days/week and 0.8 ± 1.80 days/week (See Tables 1, 2).

**Table 1:** Participant demographics.

Characteristic	*n* or m ± SD	% of sample or range
Age (year)	29.3 ± 10.0	18–77
Gender identity		
Male	149	46.0
Female	161	49.7
Non-binary or third gender	14	4.3
Race and ethnicity identity		
White or Caucasian	288	88.9
Asian	28	8.6
Other	16	4.9
Hispanic ethnicity	23	7.1
Education		
Bachelor's degree or higher	231	71.3
Children	38	11.7
Annual income		
≤$19,999	96	29.6
$20,000–$59,000	105	32.4
≥$60,000	123	38.0

*N*, number; m, mean; SD, standard deviation.

**Table 2:** Participant climbing-related characteristics.

Characteristic	*n* or m ± SD	% of sample or range
Years of climbing		
≤1 year	85	26.2
2–5 years	144	44.4
≥6 years	95	
Preferred climbing type		
Boulder	149	46.0
Top-rope	99	30.6
Sport	58	17.9
All other styles (traditional, ice, mixed)	18	5.6
Climbing level		
Novice	60	18.5
Intermediate	220	67.9
Advanced	44	13.6
Number of indoor climbing sessions per week	3.0 ± 1.7	0–14
Number of outdoor climbing sessions per week	0.8 ± 1.8	0–14

*N*, number; m, mean; SD, standard deviation.

### 3.2 Social media behaviors

Many participants report using “social media for climbing inspiration” (78.4%), follow professional climbing influencers on social media (65.7%), and the average number of social media platforms participants used was 2.76 ± 2.07. Participants reported using Instagram (78.4%) and YouTube (67.3%) most frequently for climbing-related content.

#### 3.2.1 Technology-based SCFS

The five items used from the MEIS-SCFS subscale had a Cronbach's alpha of 0.86. The mean MEI-SCFS score among all participants was 10.22 ± 4.38 (5–25). Mean MEI-SCFS outcomes were compared against multiple group variables (i.e., demographics, climbing characteristics, body type ideals, training and nutrition information-seeking behaviors, and weight management behaviors) (See Table 3).

**Table 3:** Mean differences in MEI-SCFS scores.

Characteristic	MEI—SCFS score		P
	m ± SD	*χ*^2^ (df) or *U*	
Gender identity^a^			
Male	8.73 ± 3.57	32.44 (2)	<0.001
Female	11.59 ± 4.60		
Non-binary or third gender	10.21 ± 4.79		
Age^b^			
18–24 years	11.15 ± 4.77	10,161.00	0.006
≥25 years	9.65 ± 4.04		
If my body proportions were different, then I could perform better at rock-climbing^b^			
True	11.08 ± 4.48	9,003.00	<0.001
False	8.97 ± 3.93		
Frequency of using Instagram for nutrition information^a^			
0–1×/year	9.32 ± 4.06	32.26 (2)	<0.001
2×/year—1×/month	11.68 ± 4.26		
>1×/month—daily	12.81 ± 4.67		
Frequency of using Instagram for training information^a^			
0–1×/year	8.83 ± 3.96	31.84 (2)	<0.001
2×/year—1×/month	10.95 ± 3.99		
>1×/month—daily	11.63 ± 4.61		
Follow professional rock climbers, climbing influencers or peers on social media^a^			
Yes	10.96 ± 4.40	8,151.50	<0.001
No	8.79 ± 4.00		

m: mean; SD: standard deviation.

^a^ Independent samples Kruskal-Wallis Test.

^b^ Independent samples Mann-Whitney *U*-Test.

### 3.3 Body type ideals

Many participants (66.4%) indicated strength-to-weight ratio and body type (36.1%) were moderately to extremely important for climbing performance compared to the others who perceived these characteristics as “not at all important” to “slightly important”. Many participants perceived they could perform better at rock climbing if their body proportions were different (59.3%).

#### 3.3.1 Social physique anxiety

The 11 items from the SPAS were found to have a Cronbach's alpha of 0.80. The mean SPA score was 28.75 ± 4.54 (11–43). Mean SPA outcomes were compared against multiple group variables (i.e., demographics, climbing characteristics, body type ideals, training and nutrition information-seeking behaviors, and weight management behaviors). Mean SPA outcomes were significantly different when compared to the frequency (0–1×/year, 2×/year-1×/month, or >1×/month) of using Instagram for nutrition information [*χ*^2^  =  4.93 (2,324), *p*  =  0.01]. Pairwise comparisons indicate those who use Instagram 0–1×/year had significantly lower SPA scores than those who used 2×/year-1×/month and >1×/month.

### 3.4 Climbing training and nutrition information seeking behaviors

Most participants (59.0%) ranked “pro climbers with a certification in personal training” as the most qualified to provide training information, although fewer (38.3%) have sought a professional to improve climbing performance. Most participants (80.6%) ranked “registered dietitians” as the most qualified to provide nutrition information, however only 16.1% reported seeking a registered dietitian more than once a year, and 79.3% reported “I research on my own about how to nourish my body for health or performance”. Among social media sources, participants reported using YouTube and Instagram most frequently for training and nutrition information, however, these platforms were used for training information more frequently (See Table 4). Lastly, a higher proportion of advanced climbers used YouTube for training information more frequently (>1/month—daily) than novice and intermediate climbers [*χ*^2^ = 12.84 (4,324), *p* = 0.01].

**Table 4:** Frequency of using social media sources for training and nutrition information seeking.

Source of Information	Frequency of seeking training information	Frequency of seeking nutrition information
YouTube	52.3% 4×/year—daily	18.3% 4 times/year—daily
Instagram	44.4% 4×/year—daily	22.8% 4 times/year—Daily

### 3.5 Weight management and food tracking behaviors

Almost half (42.6%) of the participants have attempted to lose weight to help their climbing abilities. Among those reporting weight loss for climbing, 73.2% report attempting to lose weight for climbing purposes four times per year or less, and 22.5% of them sought professional help to lose weight. Among all participants, only 13.5% sought professional help to lose weight and almost half (43.5%) report weighing themselves on a scale daily to once per month. Few participants (29.6%) report using food tracking methods (i.e., food journaling or food tracking apps), and most (82.1%) report using food tracking methods once every six months or less.

Logistic regression models were performed for three separate weight management and food tracking behaviors. Table 5 outlines the model for predicting the weight management behavior “attempted weight loss for climbing purposes”. Significant predictors of attempting weight loss for climbing purposes included perceiving body proportions and strength to weight important for climbing, MEI-SCFS and SPA scores, and doing one's own research on how to nourish for health and performance. Seeking professional help to reach climbing goals was borderline significant (*p *= 0.05).

**Table 5:** Logistic regression model—predictors of attempting weight loss for climbing purposes (Yes = 1; 0 = No).

Predictor	*B*	SE	Wald (χ^2^)	df	*p*	Odds ratio
Intercept (Constant)	−9.43***	1.80	27.48	1	<.001	
Age	−.51**	.29	3.03	1	.08	.60
Gender identity (male = 0, female = 1)	−.16	.31	.25	1	.62	.86
Body proportions are important for climbing (0 = Unimportant, 1 = Important)	1.40***	.29	22.67	1	<.001	4.05
Strength to weight is important for climbing (0 = Unimportant, 1 = Important)	.84*	.34	6.04	1	.01	2.33
MEI-SCFS score	.15***	.04	15.23	1	<.001	1.16
SPA score	.19**	.06	10.665	1	.001	1.21
I research on my own about how to nourish my body for health and performance (0 = Yes,1 = No)	.84*	.41	4.34	1	.04	2.33
Seek registered dietitian for nutrition advice (0 = Yes,1 = No)	.18	.28	.41	1	.52	1.19
Use Instagram for nutrition information (0 = >1/month, 1 = <1/month)	−.02	.45	.002	1	.97	.98
Use YouTube for nutrition information (0 = >1/month, 1 = <1/month)	−.02	.47	.001	1	.97	.98
Seek professionals for climbing goals (0 = Yes,1 = No)	.56	.28	3.82	1	.05	1.74
Use Instagram for training information (0 = >1/month, 1 = <1/month)	−.23	.33	.47	1	.49	.800
Use YouTube for training information (0 = >1/month, 1 = <1/month)	.29	.34	.72	1	.40	1.33

The overall model was found to be statistically significant [χ^2^ (12) = 81.99, *p* < .001], with Nagelkerke R-squared value of.31.

* *p *< 0.05.

** *p* < 0.01.

*** *p* < 0.001.

Other models inputted the same independent variables against the dependent variables: (1) “Sought professional for weight loss” and (2) “I use food tracking methods” (See Tables 6, 7). Significant predictors of seeking a professional for weight loss included perceiving body proportions and strength-to-weight important for climbing, doing one's own research on how to nourish for health and performance, and seeking a registered dietitian for nutrition advice. The overall model was found to be statistically significant [*χ*^2^ (13) = 58.08, *p* < 0.001], with Nagelkerke R-squared value of 0.25. Significant predictors of using food tracking methods included perceiving body proportions and doing one's own research on how to nourish for health and performance. The overall model was found to be statistically significant [*χ*^2^ (13) = 46.61, *p* < 0.001], with Nagelkerke R-squared value of 0.26.

**Table 6:** Logistic regression model—predictors of seeking a professional for weight loss.

Predictor	*B*	SE	Wald (χ^2^)	df	*p*	e*^β^* (odds ratio)
Intercept (Constant)	−3.27	2.40	1.86	1	.17	
Age	−.01	.02	.21	1	.65	.99
Gender identity (male = 1, female = 0)	.02	.43	.003	1	.96	1.02
Body proportions are important for climbing (1 = Unimportant, 0 = Important)	−1.03*	.45	5.34	1	.02	.36
Strength to weight is important for climbing (1 = Unimportant, 0 = Important)	−1.20*	.58	4.27	1	.04	.30
MEI-SCFS score	.07	.05	2.04	1	.15	1.07
SPA score	.09	.07	1.39	1	.24	1.09
I research on my own about how to nourish my body for health and performance (0 = Yes,1 = No)	−2.28*	1.06	4.65	1	.03	.10
Seek registered dietitian for nutrition advice (0 = Yes,1 = No)	−1.15**	.40	8.23	1	.004	.15
Use Instagram for nutrition information (0 = >1/month, 1 = <1/month)	.59	.61	.93	1	.34	1.80
Use YouTube for nutrition information (0 = >1/month, 1 = <1/month)	−.89	.56	2.54	1	.11	.41
Seek professionals for climbing goals (0 = Yes,1 = No)	−.42	.38	1.21	1	.27	.66
Use Instagram for training information (0 = >1/month, 1 = <1/month)	.02	.43	.002	1	.97	1.02
Use YouTube for training information (0 = >1/month, 1 = <1/month)	.31	.44	.48	1	.49	1.36

The overall model was found to be statistically significant [χ^2^ (13) = 46.61, *p* < .001], with Nagelkerke R-squared value of .26.

* *p *< 0.05.

** *p* < 0.01.

*** *p* < 0.001.

**Table 7:** Logistic regression model—predictors of using food tracking methods .

Predictor	*B*	SE	Wald (χ^2^)	df	*p*	e^β^ (odds ratio)
Intercept (Constant)	−3.04	1.85	2.71	1	.10	
Age	.02	.02	1.26	1	.26	1.02
Gender Identity (male = 1, female = 0)	.37	.32	1.38	1	.24	1.45
Body proportions are important for climbing (1 = Unimportant, 0 = Important)	.86**	.31	7.66	1	.006	2.37
Strength to weight is important for climbing (1 = Unimportant, 0 = Important)	.41	.35	1.33	1	.25	1.50
MEI-SCFS score	−.06	.04	2.51	1	.11	.94
SPA score	.07	.06	1.63	1	.20	1.08
I research on my own about how to nourish my body for health and performance (0 = Yes,1 = No)	1.74**	.56	9.88	1	.002	5.72
Seek registered dietitian for nutrition advice (0 = Yes,1 = No)	.53	.29	3.39	1	.07	1.70
Use Instagram for nutrition information (0 = >1/month, 1 = <1/month)	.33	.44	.56	1	.46	1.39
Use YouTube for nutrition information (0 = >1/month, 1 = <1/month)	.49	.46	1.15	1	.28	1.63
Seek professionals for climbing goals (0 = Yes,1 = No)	.18	.29	.34	1	.56	.82
Use Instagram for training information (0 = >1/month, 1 = <1/month)	−.20	.34	1.26	1	.26	1.46
Use YouTube for training information (0 = >1/month, 1 = <1/month)	.38	.34	1.26	1	.20	1.36

The overall model was found to be statistically significant [χ^2^ (13) = 58.08, *p* < .001], with Nagelkerke R-squared value of .25.

* *p *< 0.05.

** *p* < 0.01.

*** *p* < 0.001

Across each model, the body type ideal of perceiving body proportions important for climbing remained a significant predictor. The nutrition information-seeking behavior “I research on my own about how to nourish my body for health and performance” was a significant predictor for both seeking a professional for weight loss and using food tracking methods.

## 4 Discussion

To the authors’ knowledge, this was the first study to assess and test the relationship between social comparison on social media, social physique anxiety, health information-seeking behaviors, and weight management behaviors of recreational climbers. Overall, many recreational climbers in this study perceived that weight, strength, and body type were important for climbing ability. These body type ideals were related to multiple outcomes like social physique anxiety, social comparison behaviors on social media, nutrition and training-seeking behaviors, and weight management behaviors.

One theory that may explain these relationships is the Tripartite Influence Model (TIM). This model suggests that various sources (i.e., peers, family, and media) may influence the internalization of a thin ideal body image and increase risks for body dissatisfaction and compensatory behaviors (26). Recent research with young adult females (18–30 years old) demonstrated a positive linear relationship between social media use and body dissatisfaction, and appearance comparison behaviors negatively moderate this relationship (27). Similarly in our study being younger, performing more social comparisons and feedback seeking behaviors on social media and higher social physique anxiety significantly predicted attempting weight loss for climbing purposes (See Table 5). It should be noted that the mean SPA score of our sample was similar to that of other athlete and active populations (28, 29).

Almost half of our sample report attempting weight loss to improve climbing ability, which is a similar frequency to a sample of elite competition climbers (30). It should be noted that our survey did not assess details of weight loss or management practices (e.g., frequency, amount, method) or food tracking behaviors, so it is possible weight loss efforts were done safely or food tracking was for reasons other than weight management. However, our findings do note that few (13.5%) sought professional support for weight loss, which is concerning given adolescent and adult recreational and elite rock climbers fail to meet their predicted energy requirements through diet as well (1, 31). Therefore, it is possible that recreational climbers may be experiencing LEA or Relative Energy Deficiency in Sport (REDs) with or without weight loss attempts. Body type ideals like “body proportions” or “strength-weight ratio” and physique anxiety were consistently significant predictors of performing weight management and food tracking behaviors among our sample (See Tables 6, 7). Similar body ideals (i.e., drive for thinness or fitness) and use of food tracking apps have been characterized in prior research and linked to body dissatisfaction, disordered eating, and compulsive exercise behaviors among general and athletic populations (21, 32, 33).

Considering extensions of the TIM theory, social media use, weight normative content on social media, and social comparison behaviors on social media may be perpetuating these ideals. Recent research indicates many Americans (∼72%) use social media sites, which is a similar frequency to our sample (34). Similarly, YouTube, Facebook, and Instagram are used most frequently, especially among younger adults. We found that over 65% of our sample followed various climbing professionals and those with higher social comparative behaviors on social media were younger (18–24 years), identified as female, followed climbing influencers, and used Instagram frequently for training and nutrition information. Although our study did not directly assess whether climbing influencers perpetuate thin or fit ideals on social media, prior research indicates weight normative messaging and content predominates social media (20) and there is a growing market for elite and professional climbers to use social media as an additional income source. Additionally, there is a dominant ideal in the climbing community that a low weight does provide benefits to climbing, and some attribute this to how climbers are portrayed in the media (15). So, it is possible that climbing-related media and social media content combined with broader social media content collectively contribute to the perpetuation of body and weight ideals among the climbing community. It should be noted that our study did not assess other pathways of influence on social comparison such as peer or family influence. This is an important consideration given coaches, peers (other competitors and successful athletes), and internet articles influence elite climbers’ supplement use and weight loss practices (28). Collectively our findings and related data demonstrate there are many influences on an active individual's rationale to pursue a specific body type and compensatory behaviors. As it relates to the recreational climbing population's risk for injury, these pathways are associated with disordered eating and eating disorders and concerning weight-loss strategies which are significant predictors of injury outcomes and incidence of injury (35, 36).

Social media sources like YouTube and Instagram were used most frequently for training and nutrition information. Similarly, recreational, and elite climbers use YouTube most frequently for medical and injury prevention or recovery content (37), indicating that video-based platforms are preferred sources of health education content among younger climbing populations. Among our sample, training information seeking was performed more frequently than nutrition information seeking (See Table 4). It is possible that the training information sought by our participants was based on injury prevention and rehabilitation given how sports performance principles and practice overlap with sports medicine (38). Despite perceiving registered dietitians as the most qualified to provide nutrition advice, our sample preferred to research on their own about how to nourish for health and performance using online resources. Recent evidence shows a sample of elite competitive climbers have low access to access to dietitians (∼18%), which is similar to findings from our study where only 16.1% have ever met with a dietitian (28). It is important to note our study did not assess what types of training or nutrition information were sought, the modalities of information sought, whether the information was evidence-based and provided by a credentialed practitioner, and whether the participants had access to a dietitian's services.

The perpetuation of weight and body ideals continues in elite and recreational climber circles despite over a decade of research indicating that anthropometric factors are less important for climbing performance than trainable performance indicators (i.e., strength or endurance) (39). One study to date has determined recreational and elite climbers have similar anthropometric characteristics, however they do differ compared to a generally active population (13). Regardless of these differences in body composition among climbing and non-climbing populations, stronger evidence indicates trainable physical characteristics (shoulder power, finger, hand, and arm strength, flexibility, etc…) are more important for climbing performance (40, 41). Despite little to no evidence indicating anthropometric characteristics predict climbing performance both elite and recreational climbers appear to continue to practice weight manipulation behaviors, which may result from body and weight ideals perpetuated by broader social norms, social media, and traditional sport performance practices.

Expert consensus on the best practices for body composition (BC) assessment in sport recommend shifting away from the traditional approaches of emphasizing BC manipulations and conducting BC assessment without prior screening and athlete consent (6, 42). These recommendations ultimately require an interdisciplinary team of sport performance and medicine professionals and nutrition services which may be less accessible to most recreationally active individuals. Also, many athletes (competitive and recreational) and their support staff (coaches, trainers) have low awareness and knowledge of REDs and its causes (43–45). Taken together, our findings and the extant literature indicate many climbing athletes attempt weight loss, do not meet their dietary energy needs, and do not seek professionals for safe weight loss or nutrition support, it is possible an equal proportion of recreational climbers may be at increased injury risk (34, 20). It should be noted that although only a low proportion of our sample sought professional guidance for weight loss or dietitian services, we cannot discern whether this access or utilization is low compared to other climbing or non-climbing populations. Little data exists on the prevalence of non-athletic populations access or use of dietitian services. In one study, general practitioners referred 0.26% of patients with a nutrition-related problem/diagnosis (diabetes, overweight/obesity) to a dietitian (46). In a hospital setting, overall referral rate was ∼17% (47). No relevant data exists on the prevalence of athletic population access to dietitian services, however, nutrition services at collegiate and professional levels appears to be growing and has shown to improve nutrition knowledge and related outcomes like adequate calorie and macronutrient intakes (48). Ultimately, a broader understanding of recreational climbers’ access and use of sport performance and dietitian services, as well as their nutrition or weight management needs can help advance access to education and resources designed to prevent LEA and REDs.

## 5 Limitations

Because this specific climbing population and its unique social and economic characteristics have not been characterized in prior research, measures not previously tested were developed and included in the analysis. The reliance on self-reported measures and lack of prior testing on these un-validated measures may limit the accuracy and reliability of the findings (49, 50). The benefit of piloting these variables provides insight for future researchers to develop and test validated measures and tools to assess climbing populations’ health risks. It should also be noted that respondents may be hesitant to truthfully respond to sensitive measures related to social physique anxiety, social comparison and feedback seeking on social media, and weight loss (51, 52).

Another possible weakness of the study is that by distributing to indoor climbing gyms, we excluded climbers who predominantly climb outdoors which may influence various training, nutrition, and weight-related behaviors. However, most of the climbers in our sample climbed outdoors in addition to climbing indoors. Also, we did not directly assess climbing grade to determine a more objective climbing ability (53), rather we assessed a general self-reported perception of climbing level (novice, intermediate and advanced). Finally, since our study did not include non-climbers or athletes of other sports, this limits our ability to discern whether behaviors and associated outcomes differ from non-climbers or other athletes. Stronger evidence can be found in future studies that make these comparisons or use randomized controlled trials.

## 6 Conclusion and Recommendation

Our research indicates that recreational rock climbers are not immune to the body or weight-based pressures felt by elite-level or competition climbers. It also suggests that social comparison on social media mediates the internalization of thin and fit ideals as they relate to climbing. Given these internalizations may lead to compensatory behaviors like dieting or compulsive exercise to change body weight, which may lead to increased injury risk, future research should explore these relationships and their potential interventions among recreational climbers.

Future research can assess the various roles of family, peers, climbing influencers, and climbing media in perpetuating fitness and body type ideals in the climbing community. These findings could support the development and testing of evidence-based interventions that promote weight-inclusive training and nutrition practices and messaging to affirm participants of various shapes and sizes and remove the inclination that participants must have ideal body types to be successful at climbing. Ultimately, more weight-neutral and body-positive interventions and evidence-based training and nutrition resources for the climbing community could help climbers develop healthy relationships with climbing, and their bodies, and decrease the likelihood of injury.

## Data Availability

The raw data supporting the conclusions of this article will be made available by the authors, without undue reservation.
